# Spectrum of Interventional Procedures During Hybrid Central Line Placement in Pediatric Intestinal Rehabilitation Patients With End-Stage Vascular Access

**DOI:** 10.3389/fnut.2022.863063

**Published:** 2022-03-28

**Authors:** Ludger Sieverding, Jörg Michel, Christian Urla, Ekkehard Sturm, Franziska Winkler, Michael Hofbeck, Jörg Fuchs, Johannes Hilberath, Steven Walter Warmann

**Affiliations:** ^1^Department of Pediatric Cardiology, University Children's Hospital, University of Tübingen, Tübingen, Germany; ^2^Department of Pediatric Surgery and Pediatric Urology, University Children's Hospital, University of Tübingen, Tübingen, Germany; ^3^Department of Pediatric Gastroenterology and Hepatology, University Children's Hospital, University of Tübingen, Tübingen, Germany

**Keywords:** pediatric intestinal failure, parenteral nutrition, thoracic central venous obstruction, hybrid central venous access, revascularization, thrombectomy, angioplasty, vascular rehabilitation

## Abstract

**Background:**

Loss of available central vein access sites for parenteral nutrition delivery represents one of the main indications for intestinal transplantation in children with intestinal failure. Placement of central venous catheters can be challenging in advanced loss of patent venous pathways. We recently described the hybrid technique (interventional plus surgical approach) of central line placement in children. The aim of this study was to describe and analyze the interventions used during the hybrid procedures regarding feasibility, safety and outcome.

**Methods:**

We retrospectively analyzed the course of all children in our intestinal rehabilitation program undergoing hybrid central line placement. We evaluated patients' conditions, interventional techniques and surgical peculiarities as well as outcome.

**Results:**

203 children were treated in our intestinal rehabilitation program between 2010 and 2021. Due to loss of venous access, hybrid technique was performed in 53 children during 76 interventions. In 40 cases the same vessel was reused *via* Seldinger technique. Among the 30 ultrasound-guided new vessel punctures, 12 were performed by puncture of collateral vessels. Extended interventions due to thoracic central venous obstruction and/or thrombosis requiring additional access *via* a femoral vein for rehabilitation of the vascular system was performed during 29 procedures including catheter extraction (1), angioplasties (18), stent placement (1), revascularization (5) and thrombectomy (4). Placement of a central line was not possible in 6 children which eventually underwent extended thoracic/vascular surgery: in three children the previously placed catheter could not be removed, in one child, placement of a thrombectomy-catheter was not possible because of inferior vena cava occlusion, and in two children, revascularization failed. Intestinal transplantation was considered in one patient because of impending loss of vascular access. Two self-limiting minor extravasations and one intervention-associated pericardial effusion occurred.

**Conclusions:**

Hybrid interventions for central venous catheter placement and vascular rehabilitation enable a high success rate in children with intestinal failure and end-stage vascular access, circumventing the need for intestinal transplantation or advanced surgery. The relevant procedures are complex and require a foresighted and individualized approach with a wide range of interventional techniques. If performed with expertise, this combined interventional/surgical approach is feasible and safe.

## Background

Multidisciplinary management within intestinal rehabilitation programs (IRP) is regarded as the current standard for treatment of children with chronic intestinal failure (1, 2). These interdisciplinary programs address the complexities of affected patients by managing their special needs, anticipating complications and providing coordinated and individualized care. For pediatric patients with intestinal failure, IRPs have been found to have a significant impact on morbidity as well as demonstrating improvement in survival (3) and allow in many cases a better long-term outcome compared to alternative therapies such as intestinal transplantation. Those advances in the Non-transplant management of intestinal failure led to a reduction in the number of intestinal transplantations worldwide from a peak of 270 per year in 2008 to 149 per year in 2017 (4). However, various risks still remain for affected children, a relevant number of them being associated with the inevitable permanent central venous lines. Infection, dislocation or malfunctions of the central catheters frequently make removal and/or replacement of catheters necessary. Over time, children have a relevant risk for loosing vessels suitable for catheter placement (5, 6). In cases of end stage vascular access with thoracic central venous obstruction (TCVO), placement of permanent central venous catheters may be challenging and is frequently deemed impossible. Therefore, the loss of central vascular access represents one of the main reasons for the need of intestinal transplantation in children with chronic intestinal failure. A consensus paper from 2019 defined thrombosis of 3 out of 4 discrete upper body central veins (left subclavian and internal jugular, right subclavian and internal jugular) or occlusion of a brachiocephalic vein in children as one criterion for placement on a waitlist for intestinal transplantation (4). We recently described the successful use of hybrid central line placement (interventional plus surgical approach) in affected children (7). Essentially, the procedure consists of 2 basic techniques. First, the replacement of previously placed central vein catheters *via* an exchange wire and a split cannula in Seldinger technique. The other is the ultrasound-guided puncture of central veins or in case of severe vessel loss puncture of thin-walled, often tortuous collateral vessels, through which flexible, ultra-thin interchangeable wires and microcatheters can be passed until the superior vena cava (SVC) is reached. Both techniques are combined with additional interventional procedures for vascular rehabilitation when necessary. The aim of this strategy is to avoid a change of vascular access site, to detect and prevent impending vascular occlusions at an early stage, to recanalize thrombosed vessels or to establish a new pathway. The primary aim of the present study is the analysis of all hybrid procedures performed at our center between 2010 and 2021 with regard to feasibility, safety, efficacy and outcome. Secondary objective is to describe the techniques used during hybrid interventions.

## Patients and Methods

We retrospectively analyzed the course of all hybrid intervention procedures for central line placement performed between 2010 and 2021 in children with chronic intestinal failure within our pediatric intestinal rehabilitation program. All children underwent ultrasound scan of their vascular system. If thromboses or stenoses of a large vein were identified or if the vascular status of the upper central veins could not be clarified *via* ultrasound, children underwent MR-angiography or CT-angiography. The thoracic central vein obstructions (TCVO) were classified according to the recommendation of the International Society of Interventional Radiology (Table 1) (8). The decision to perform catheter placement as hybrid intervention was taken on an interdisciplinary platform based on the clinical course of children so far, especially regarding their vascular conditions at the time of evaluation for catheter placement. Evaluation of the diagnostic workup as well as determination of the interventional strategy was undertaken in consensus reading between a pediatric surgeon, pediatric cardiac interventionalist, pediatric gastroenterologist and pediatric radiologist.

**Table 1:** Classification of thoracic central venous obstruction (TCVO) (8).

Type 1:	Both BCVs and the SVC are patent, but one IJV or SCV is obstructed. If patency of all thoracic veins cannot be determined, this type of TCVO can be classified as type 1A or type 1B.
*Type 1A:*	*Unilateral IJV or SCV obstruction with patent ipsilateral BCV; patency of all other thoracic central venous anatomy is not known*.
*Type 1B:*	*Unilateral IJV or SCV obstruction with patent ipsilateral BCV; patency of contralateral thoracic central venous anatomy is not known*.
*Type 1C:*	*Unilateral IJV or SCV obstruction with known patency of the contralateral IJV, SCV, and BCV*
*Type 1D:*	*Bilateral obstruction of IJVs, SCVs, or combined IJV and SCVs, with both BCVs patent*.
Type 2:	Any form of TCVO that causes unilateral BCV obstruction or ipsilateral obstruction of the IJV and SCV (equivalent to unilateral BCV obstruction)
*Type 2A:*	*Unilateral BCV obstruction with unknown condition of the contralateral side*
*Type 2B:*	*Unilateral BCV obstruction with known patency of the contralateral side*.
Type 3:	Both BCVs are obstructed, but flow to the right atrium passes through the SVC.
Type 4:	SVC obstruction that prevents or impedes direct thoracic venous flow to the right atrium with any constellation of BCV, IJV, or SCV obstruction

*BCV, brachiocephalic vein; IJV, internal jugular vein; SCV, subclavian vein; SVC, superior vena cava*.

All hybrid interventions were performed under general anesthesia. Depending on the patients' size a 4.2 French (Fr.) or 6.6 Fr. single lumen permanent central venous catheter (Broviac^®^ catheter, C.R.Bard GmbH, Karlsruhe Germany) was implanted. Children received a single shot antibiotic prophylaxis intravenously at the beginning of intervention.

In case of replacement of a previously inserted catheter (with the catheter whether displaced or not), the catheter was surgically dissected at the site of entrance into the vessel and was then cut through. The distal limb was secured using a retaining suture and a 0.018 “exchange wire was inserted. Following extraction of the distal limb a 4 Fr. sheath was then initially introduced *via* the exchange wire. Through this sheath, a 4 Fr. catheter was advanced to inferior vena cava (IVC). A stiff 0.035” exchange wire was inserted into the inferior vena cava *via* the catheter. The vessel was then expanded with the aid of vessel dilatators of ascending size followed finally by the insertion of a 7 Fr. split cannula. Once the split cannula was in place, the placement of the new permanent central catheter was performed surgically according to the classical technique including skin incision, subcutaneous tunneling, and intravascular placement through the split cannula.

Placements of new catheters were performed *via* ultrasound-guided puncture of a central vein or an accessible collateral vessel followed by the same procedure as described above.

If necessary, extended interventional procedures were applied to reestablish or improve a pathway for catheter placement. Vascular management in such cases included various techniques such as revascularization, balloon angioplasty, stent implantation or thrombectomy. If required angiography and/or interventional procedures were performed using the access *via* the femoral vein and IVC.

Data were assessed with special focus on interventional techniques used during the procedures. We evaluated patients' conditions, interventional and surgical peculiarities, as well as outcome of the hybrid placement of central venous catheters.

### Statistical Analysis

**Figure 1:**
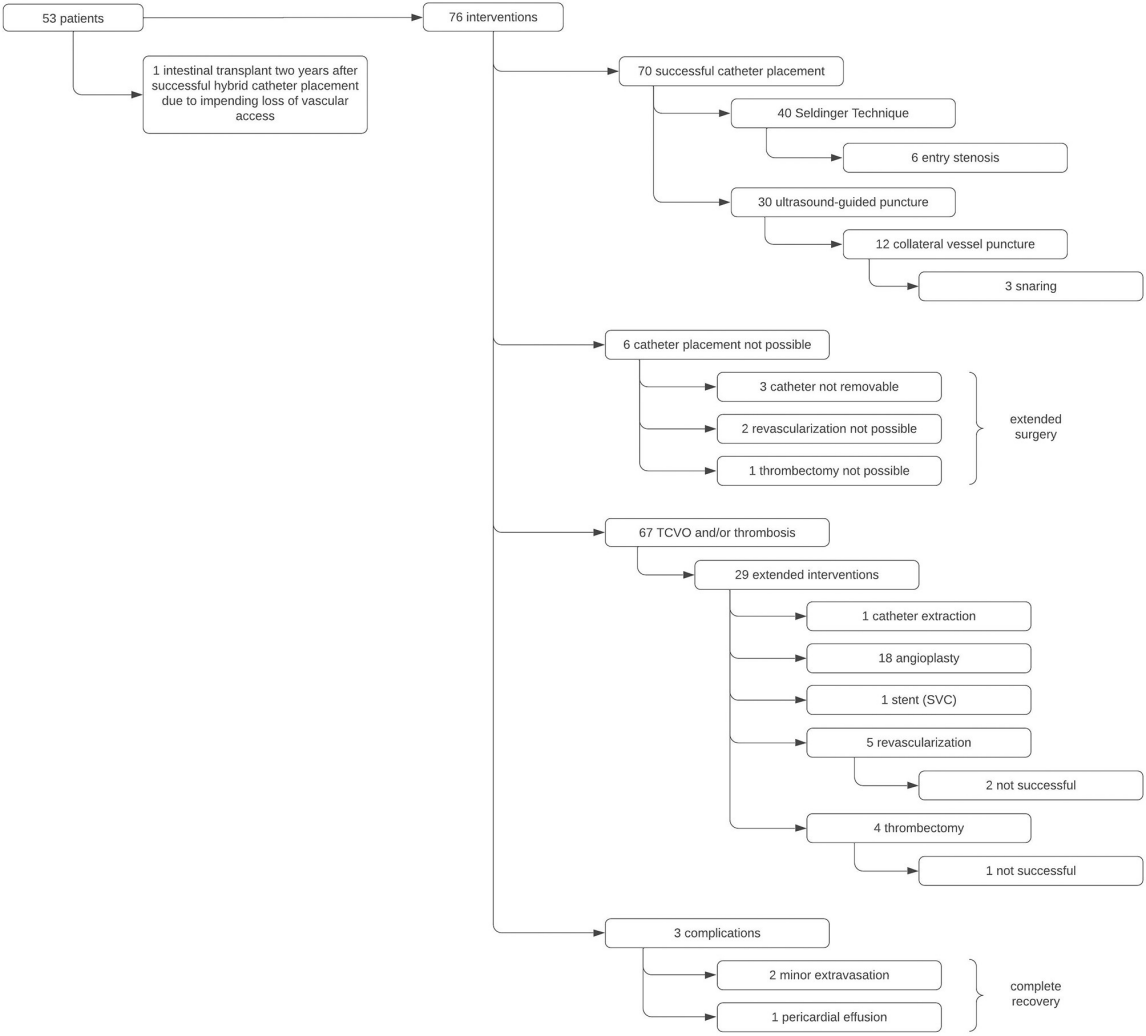
Overview of the interventions performed during hybrid central line placement, see text for details. TCVO, thoracic central venous obstruction; SVC, superior vena cava.

Data analysis was performed using the JMP^®^ 11.2 statistical software (SAS Institute, Cary, NC). Quantitative data are summarized with the median, interquartile range (IQR), and minimum and maximum values. Figures 1, **5** were created in Lucidchart (www.lucidchart.com).

## Results

Seventy six interventions were performed in 53 patients (29 females, 24 males) with chronic intestinal failure (Figure 1). Indications for catheter placement included catheter dislocation, malfunction, disruption or reinsertion following removal due to infection. The number of previously inserted central venous catheters (CVC) ranged from 1 to 14.

Patients and interventions characteristics are listed in Tables 2–4.

**Table 2:** Patient characteristics (*n* = 53).

**Sex (m/f)**	***n* (%)**	**24 / 29 (45.3% / 54.7%)**
Short bowel syndrome	n (%)	38 (71.7%)
*Agenesis/Atresia/Stenosis*	*n (%)*	*5 (9.4%)*
*Necrotizing enterocolitis*	*n (%)*	*12 (22.6%)*
*Gastroschisis*	*n (%)*	*11 (20.7%)*
*Volvulus*	*n (%)*	*9 (16.9%)*
*Long segment Hirschsprung's disease*	*n (%)*	*2 (3.8%)*
Pediatric intestinal pseudoobstruction (PIPO)	n (%)	7 (13.2%)
*Megacystis microcolon intestinal*
*hypoperistalsis syndrome*	*n (%)*	*1 (1.9%)*
*Hypoganglionosis*	*n (%)*	*3 (5.7%)*
*Sec. PIPO following ischemia*	*n (%)*	*1 (1.9%)*
*Sec. PIPO following infection*	*n (%)*	*1 (1.9%)*
*Idiopathic*	*n (%)*	*1 (1.9%)*
Mucosal enteropathy	n (%)	7 (13.2%)
Microvillus inclusion disease	n (%)	6 (11.3%)
Tufting enteropathy	n (%)	1 (1.9%)
Malabsorption after stem cell transplant	n (%)	1 (1.9%)

**Table 3:** Number of interventions (*n* = 76) per patients (*n* = 53).

	***N* (patients)**	**%**	***N* (interventions)**	**%**
1 intervention	38	71.7	38	50
2 interventions	10	18.9	20	26.3
3 interventions	4	7.5	12	15.8
6 interventions	1	1.9	6	7.9
Total	53		76	

**Table 4:** Intervention related characteristics (*n* = 76).

**Intervention**	**Median**	**Range**	**IQR**
Age (years)	5.05	0.208–21.03	2.9–8.4
Weight (kg)	15.05	3–58	1.6–21
Body Surface (m^2^)	0.64	0.19–1.68	0.53–0.82
Catheter time (min)	81	15–341	50.75–117.5
Irradiation time (s)	229	24–3,961	117–734.8
Irradiation dose (Gycm^2^)	0.425	0.03–12.31	0.16–1.308
Contrast media (ml/kg)	1.1	0.1–12	0.47–3.12

In summary catheter replacement in Seldinger technique was possible in 40 procedures and catheter placement by ultrasound guided vessel puncture in 30 investigations. Placement of the central venous line was impossible in 6 cases (3 failed catheter removal, 1 failed thrombectomy due to complete occlusion of the infrarenal IVC, 2 failed revascularization of left brachiocephalic vein). These 6 children eventually underwent extended thoracic/vascular surgery. During the study period none but one of the patients had to undergo intestinal transplantation due to impending loss of vascular access.

### Collaterals Vessels

Among the 30 new punctures, 12 were performed by ultrasound-guided puncture of collateral vessels. Under fluoroscopic control, a floppy guidewire could be advanced to the superior vena cava in 9 patients. In 3 patients, the guidewire deviated into other vessels. After transfemoral snaring, the guide wire could be positioned in the superior vena cava.

**Figure 2:**
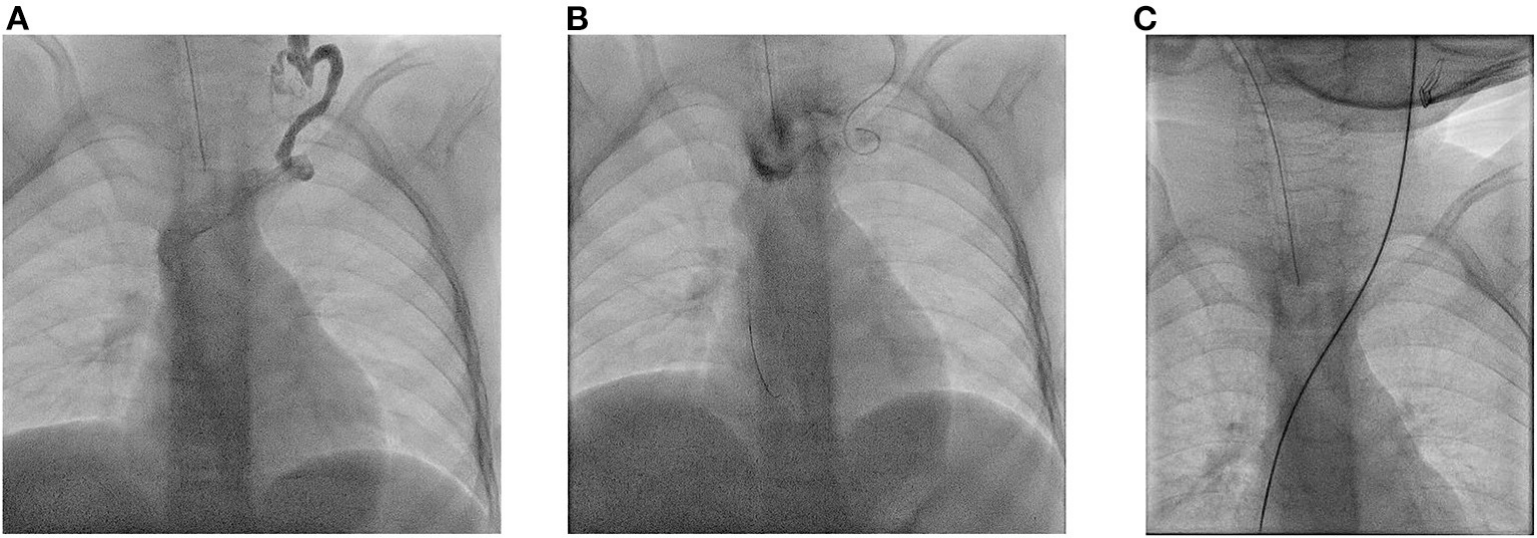
Three years old girl with obstruction of right jugular vein, right subclavian vein, right brachiocephalic vein and left jugular vein. **(A)** After puncture of a left collateral vein contrast injection shows the tortuous course of the vein entering the left brachiocephalic vein. **(B)** After probing of the collateral a superfloppy guidewire has been advanced into the superior vena cava **(C)** After probing with a catheter and insertion of a stiff wire, the collateral vessel has been stretched over its entire length.

Despite the usually very tortuous course of the collateral vessels, insertion of a 7 Fr. split sheath was possible in all cases, since these vessels stretch after insertion of a guide catheter and a stiff guide wire (Figure 2). After removal of the guide wire and dilator, kinking of the thin-walled split cannula occurred in 4 cases, so that advancement of the central venous catheter was not possible. Reinsertion of the guide wire resolved the kinking. The central venous catheter was then threaded onto the guide wire and advanced into the superior vena cava.

### Catheter Removal

After insertion of the exchange wire removal of the previously inserted catheter was impossible in 3 patients with immediate fragmentation of the catheter in one. In the latter, the catheter had been in place for 15 years. In this case the remaining part of the catheter was removed surgically. After interdisciplinary discussion, the catheter was left in place in the other two cases and surgical placement of a new catheter was performed.

### Stenosis of the Entry Site

After removal of the previous catheter balloon-angioplasty (1–4 mm balloon diameter) of the entry site had to be performed in 6 patients as introduction of adequately sized dilators was not possible.

### TCVO and Thrombosis

In all but 9 interventions thrombosis of at least 1 central venous catheter site was present (Table 5). As vascular obstruction was expected at the catheter site after catheter removal these patients were also included in the hybrid program. Involvement of femoral veins, iliac veins or inferior vena cava was observed in 11 patients with complete occlusion of the infrarenal IVC in 4 patients and bilateral thrombosis of the femoral or iliac veins in 4 patients. Extended procedures because of TCVO and/or thrombosis were performed in 29 interventions (18 angioplasties, 1 stent, 5 revascularizations, 4 thrombectomies and 1 catheter extraction) and required an additional interventional access *via* a femoral vein (Tables 6, 7).

**Table 5:** Characteristics of thoracic central venous obstruction (TCVO) according to the guidelines and classification of the Society of Interventional Radiology (8) (*n* = 76).

	***n***	**%**
None	9	11.8
TCVO 1C	13	17.1
TCVO 1D	9	11.8
TCVO 2b	35	46.1
TCVO 3	6	7.9
TCVO 4	4	5.3

**Table 6:** Type of interventions (*n* = 76).

	***n***	**%**
Simple procedure	47	61.8
Extended procedure	29	38.2
*Single angioplasty*	*11*	*14.5*
*Multiple angioplasties*	*7*	*9.2*
*SVC-Stent*	*1*	*1.3*
*revascularization*	*5*	*6.6*
*thrombectomy*	*4*	*5.3*
*catheter extraction*	1	1.3

**Table 7:** Number of angioplasties (*n* = 26).

	***n***
Collaterals	3
Right jugular vein	2
Right subclavian vein	1
Right brachiocephalic vein	5
Left jugular vein	6
Left brachiocephalic vein	7
SVC	1
IVC-Stent	1

### Angioplasty and Stent

26 angioplasties of central thoracic veins (Table 7) were carried out in 18 interventions (3 collaterals, 2 right jugular vein, 1 right subclavian vein, 5 right brachiocephalic vein, 6 left jugular vein, 7 left brachiocephalic vein, 1 SVC, 1 IVC-stent). Three of the stenoses of the left brachiocephalic vein were isolated stenoses at the orifice into the superior vena cava. One stenosis occurred in combination with a stenosis of the right brachiocephalic vein and the right superior vena cava, respectively. Two long-distance stenoses of the left brachiocephalic vein were present in combination with a stenosis of the left internal jugular vein.

### Revascularization

**Figure 3:**
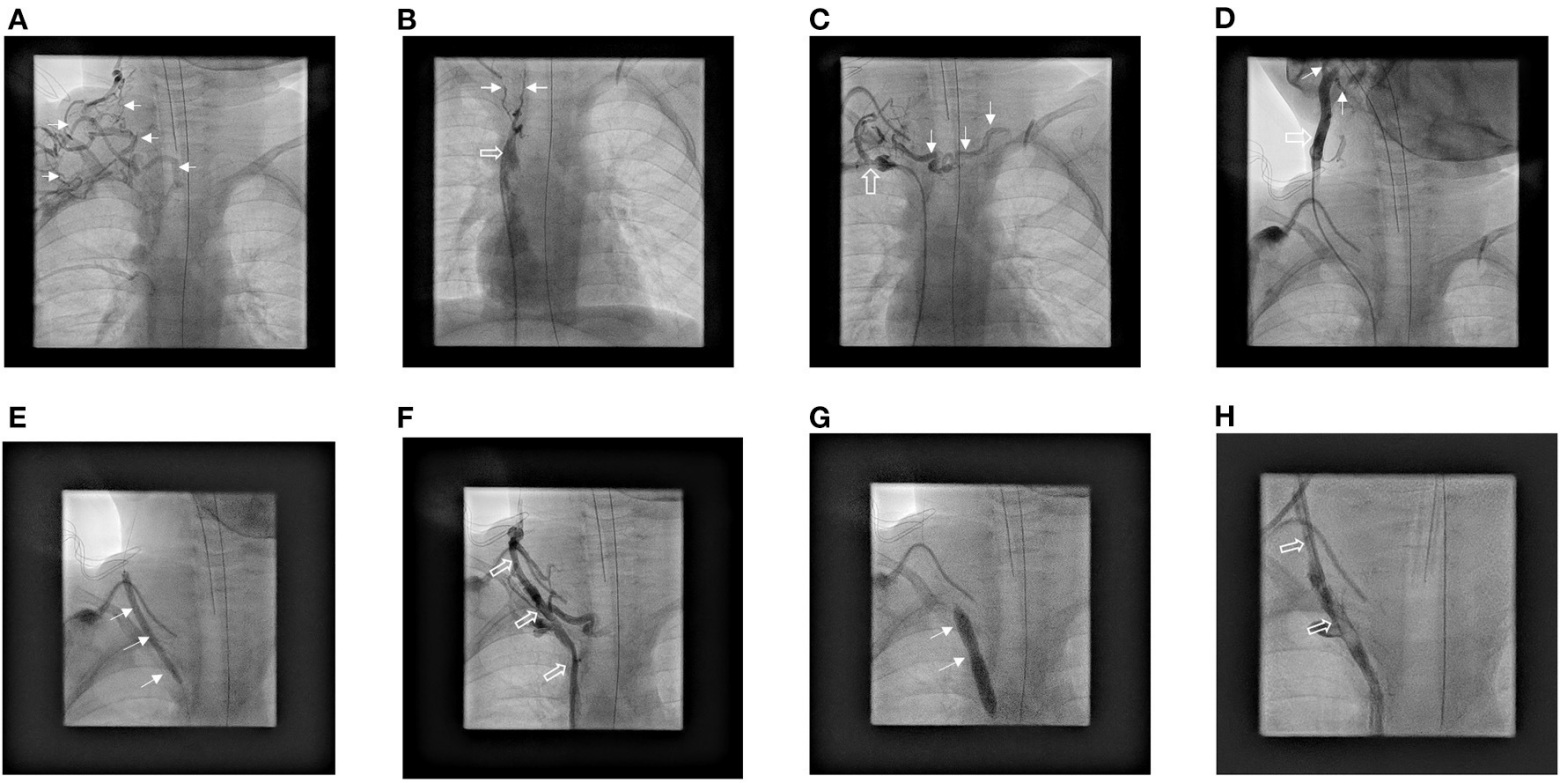
Eight years old girl with obstruction of left and right brachiocephalic vein. **(A)** After contrast injection *via* right peripheral i.v. line a diffuse collateral pattern is displayed (small arrows). **(B)** Transfemoral probing of the SVC (open arrow). After contrast medium administration, only small connecting vessels to the neck veins are stained (small arrows). **(C)** After successful probing of the subclavian vein contrast injection leads to collateral flow to the left side. **(D)** After probing of a remnant of the right jugular vein contrast injection leads to retrograde flow to the left side without any antegrade Flow to the brachiocephalic vein (small arrows). **(E,G)** Balloon angioplasty (small arrows) with increasingly larger balloons (4–7 mm) **(F,H)** restores the flow to SVC (open arrows).

In 4 patients with chronic total occlusion of the left brachiocephalic vein, we attempted to restore the connection between the superior vena cava and the left-sided neck vessels. In 2 of them the distal and proximal stump of the left brachiocephalic vein was probed in a bidirectional approach from the femoral and cubital sides, respectively, and a 1.7 Fr. microcatheter (Progreat^®^, Terumo, Shibuya, Japan) was advanced under angiographic control with an internal super floppy guidewire. However, complete passage was not achieved and the final contrast injection *via* the microcatheter resulted in a small extravasation in each case. In the other two patients, a connection to the left side of the neck could be established with the aid of a super floppy exchange wire from the superior vena cava *via* small bridging veins. Subsequent balloon angioplasties enlarged these bridging veins sufficiently. In one patient revascularization of right brachiocephalic vein was successfully completed by balloon angioplasty after passage of a microcatheter (Figure 3).

### Thrombectomy

**Figure 4:**
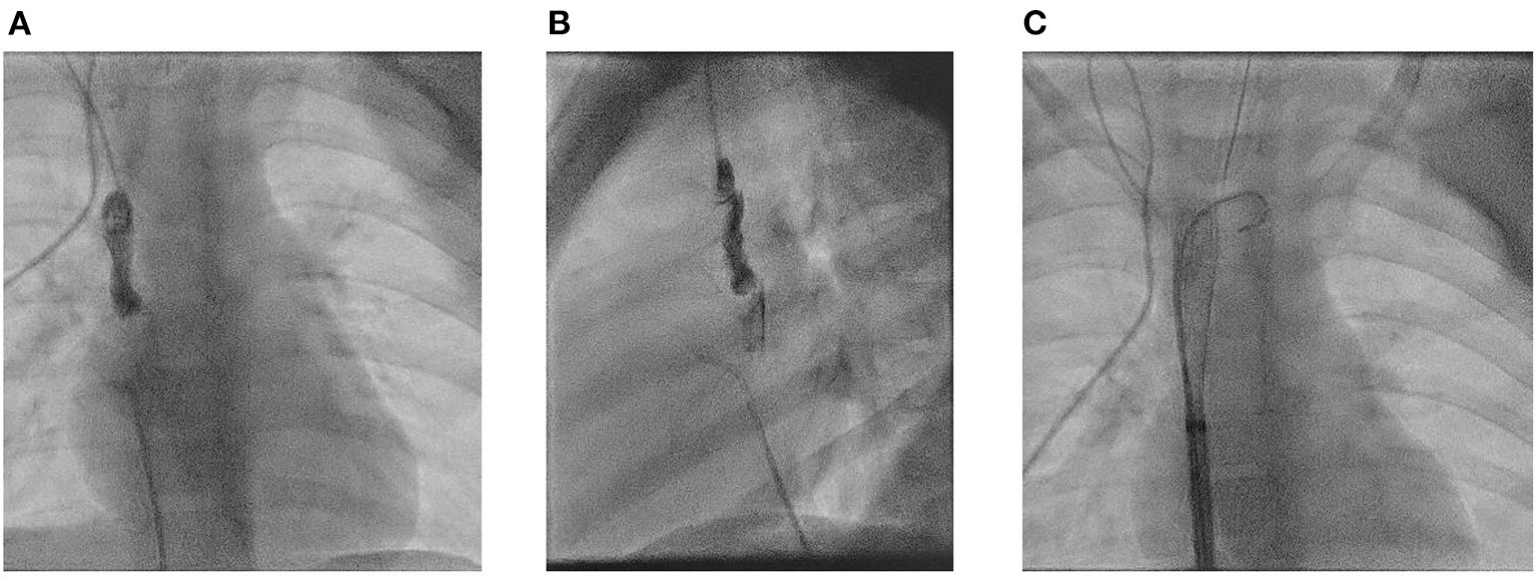
Three years old girl with a huge thrombus extending from the catheter tip to the RA. **(A)** Anterior-posterior projection. **(B)** Lateral projection. **(C)** Removal with the Capturex device^®^.

Floating thrombi at the catheter tip with the risk of embolization were present in 4 patients. In 3 patients, these could be retrieved using a catch basket catheter, in 2 of them using the Capturex® device (Straub Medical AG, Wangs, Switzerland) (Figure 4) and in one a stone basket (Urotech GmbH, Rohrdorf, Germany). Due to the occlusion of the infrarenal IVC the use of this technique was not possible in one patient.

### Complications

In addition to minor contrast extravasation in 2 patients undergoing revascularization, one patient experienced a bloody pericardial effusion due to misplacement of a stiff guidewire. Pericardial tamponade was prevented by immediate placement of a pericardial drain, and the patient recovered completely.

## Discussion

Children with chronic intestinal failure depend on permanent central venous catheters for delivery of parenteral nutrition as long as they have not achieved enteral autonomy. The knowledge about the importance of avoiding catheter-related complications has increased over recent years and has led to specific handling recommendations with regard to the central lines (7, 9–14).

In addition to careful catheter care and consistent monitoring with regard to catheter function, catheter position, catheter-associated infections, thrombi and stenoses, the strict reuse (whenever possible) of the once selected catheter access site by catheter replacement in Seldinger technique is crucial to reduce the number of catheter-associated vascular thromboses. This should be observed from the first time a central venous catheter (CVC) is inserted. Of course, the need for long-term parenteral nutrition cannot be foreseen in the early phase of the disease in many clinical cases. Nevertheless, careful monitoring for thrombosis and vascular stenosis should be carried out with every CVC in the newborn and infant. This applies in particular to CVC installations *via* the femoral veins, as thrombosis of the femoral or iliac veins or even the IVC may be clinically inapparent. However, this vascular access is essential for detailed angiographic visualization of the venous vascular situation in the area of the upper thoracic aperture, for safe placement of guide wires, for angioplasties, stent implantations and thrombectomies. Eleven of our patients had occlusions in this area with a lack of transfemoral access in 8 patients due to bilateral occlusion of the femoral or iliac veins (*n* = 4) or occlusion of the IVC (*n* = 4). Whether transfemoral catheter placement is generally associated with an increased risk of thrombosis remains controversial (15–18).

After long-term chronic vessel occlusion, revascularization is often extremely difficult and often not sustainable due to the pronounced collateral circulation. In our study revascularization was successful in only 3 out of 5 attempts. Although catheter insertion *via* collateral vessels is often still possible when central veins are occluded, thrombosis should be treated as early as possible. Particular attention should also be paid to vascular stenoses, as these are often precursors to later vascular occlusion. Accordingly, a detailed vascular diagnosis should be made before any replacement of a central venous catheter. Depending on the vascular status, an ultrasound examination of the vessels of the upper thoracic aperture may be sufficient. If vascular occlusions are already present, a cross-sectional CT and/or MRI scan should be performed before the intervention in order to be able to plan the interventional procedure with sufficient precision (19). In particular, if central access only seems possible *via* collateral vessels, high-resolution imaging of the collateral vessels is essential in order to be able to determine the most promising access route in advance. Nevertheless, in patients with end stage vascular access, supplementary venography of the venous system of the upper thoracic aperture by contrast injection *via* peripheral veins proofed extremely valuable and was performed in all our patients with this type of vascular status.

Based on the results of the previous diagnostics, a rehabilitation of the vascular system by revascularization, angioplasty, thrombectomy or stent placement was performed whenever possible for all catheter replacements or new catheter installations. This explains the occasionally long examination times, the amount of contrast media and the radiation dose. The maximum dose of contrast media was 12 ml/kg which corresponds to the maximum dose for cardiac catheterizations in children. On average, the doses of contrast medium with an IQR of 0.47–3.1 were below the doses used in cardiac catheterizations (20). In 33 examinations, no contrast medium was used at all. During the procedures a median fluoroscopy dose of 0.425 Gy^*^ cm^2^ was applied, which correlates with a median effective dose (ED) of 0.59 mSv considering the conversion factor (~1.4) under the given circumstances (filtration, tube potential, localization, age of patients) (21). The effective dose for a current standard thoracic CT scan usually lies between 1 and 2 mSv.

### Collateral Vessels

In all but 6 patients, new or exchanged central venous catheters were inserted *via* left or right neck vessels, 12 of which were collateral vessels. Only in one patient a temporary transfemoral catheter was inserted in case of complete occlusion of the upper thoracic aperture. In this patient, puncture of a collateral vessel was also successful; however, with occlusion of all jugular veins, subclavian veins, and brachiocephalic veins, drainage of the upper half of the body was *via* an accessory hemiazygos vein to the hemiazygos vein, which ultimately drained into the inferior vena cava *via* communication with the azygos vein. As this was the only remaining drainage for head, neck, and arm vessels, catheter insertion was omitted to avoid a “superior vena cava syndrome”, especially since the patient suffered from homozygous Factor V Leiden mutation. Two months later a surgical catheter implantation was performed *via* a sternotomy followed by small intestine transplantation 2 years later.

In addition, next to a possible drain obstruction attention should also be paid to the course of the collateral vessels, as these often run subcutaneously and occasionally cross the larynx, which can trigger a pronounced discomfort in patients.

When all options of jugular, subclavian, or femoral catheterization have been exhausted, as in two of our patients, the option of surgical catheter placement directly into the superior vena cava or right atrium remains. Other sites for surgical CVC placement (such as transhepatic, translumbar, or intercostal) are feasible, but all of these options are not easily performed in small children. A good compilation of these alternative vascular accesses can be found in the Chaochankit's review (22).

### Catheter Removal

In 3 patients, previously inserted catheters could not be removed because it was obviously firmly attached to the vessel wall. In one case with 15 years indwelling time, the catheter began to disintegrate with even the slightest traction. This catheter was ultimately surgically removed because it could not be ruled out that portions of the distal catheter fragment would embolize. We could not find any technical information on when to expect the material degradation of the catheters.

In the other two cases with shorter indwelling times, the catheters remained intact. Although several techniques are available to mobilize such “stuck” catheters (23, 24), catheter removal was not performed in both cases because internalization into the vessel wall had already to be assumed, and open surgical catheter removal would mean an inadequately large trauma to the vessel. This procedure corresponds to a frequent practice in pediatric patients (25, 26).

### Stenosis of the Entry Site

Although not previously described, we had difficulty inserting sheaths or dilators over the guidewire in 5 patients, probably due to significant adhesions and scarring at the catheter entry site. Dilatation of this area with the aid of small size coronary balloons eliminated this obstacle to passage.

### Angioplasty

Percutaneous transluminal angioplasty is the basic technique for treating central venous stenoses as well as venous lesions distal to the vascular access site and has been successfully applied for 26 lesions in 18 interventions. Balloon angioplasty of venous stenoses and thromboses is an established technique in pediatric interventional cardiology and interventional radiology (27–31). Studies on the systematic use of this procedure in short bowel syndrome patients are not available. The balloon diameter depends on the extent of the stenosis and should not exceed the normal expected vessel diameter to avoid dissection. In case of dissection or high-grade restenosis, stent implantation may be considered. In general, stent implantation should be avoided in this disease setting because of the problematic anticoagulation.

### Revascularization

Revascularization even in the presence of long-distance vascular thrombosis has been described (32–34). Nevertheless, when advancing catheters within a thrombosed and sclerosed vascular sheath, intramural or extravascular location of the catheter must be anticipated. The use of small bridging veins is an alternative to restore a sufficient connection between left jugular veins and the right superior vena cava. However, with marked collateral flow, there is a possibility of rapid thrombosis even in the presence of a sufficient diameter of the newly created connection.

### Thrombectomy

Three types of catheter-related thrombosis may develop, including a fibrin sheath around the catheter tip, an intraluminal blood clot, and venous thrombosis. Venous thrombosis can be either acute venous thrombosis, especially right atrial thrombosis with a high risk of pulmonary embolism or chronic venous thrombosis, which is usually clinically silent and only discovered during systematic follow-up (35, 36). In 4 of our patients, long-distance thrombi were found along the catheter with floating parts in the right atrium. With the Capturex^®^ device thrombi could be picked off the catheter and safely retrieved. However, the catheter diameter of 10 Fr. restricts the application in pediatric patients.

### Interventional Algorithm

**Figure 5:**
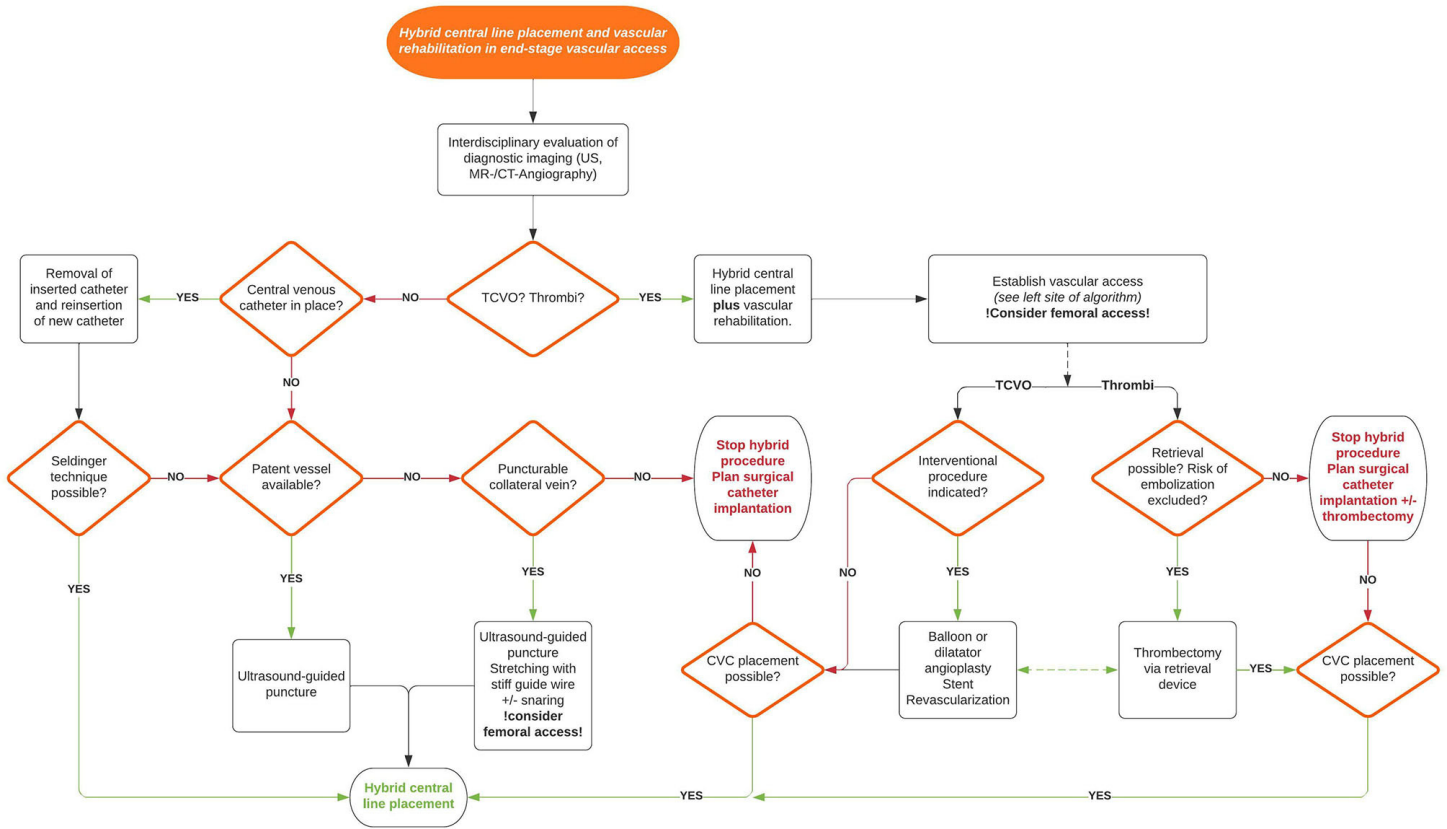
Interventional algorithm for hybrid central line placement and vascular rehabilitation in end-stage vascular access. US, ultrasound; TCVO, thoracic central venous obstruction; CVC, Central venous catheter.

The approach to the child with intestinal failure and end-stage vascular access can be very complex and individual solutions are sometimes required. Based on our data analysis and experience of more than 10 years with the hybrid intervention technique we developed an interventional algorithm reflecting different clinical scenarios (Figure 5). The goal is to provide guidance and to underline the approach to place a new catheter and provide rehabilitation of the vascular system in the same intervention whenever feasible.

### Complication

The procedure related complication rate was lower than published data for insertion related surgical implantations (3.9% vs. 7–18%) (37, 38). In addition to the 2 extravasations, 1 pericardial effusion occurred. The latter underlines the importance of a stable catheter position in the IVC before insertion of the stiff guidewire. This should also be done under strict fluoroscopic control in order to correct the catheter position if necessary.

### Study Limitations

A limitation of this study lies in the lack of a differently managed group for comparison. However, this fact is mainly caused by the nature of the clinical management of our patients. The authors are convinced that they are delivering a type of care, from which the patients largely benefit. This is furthermore underlined by the fact that despite presenting with in extremely poor vascular conditions, only one patient had to undergo intestinal transplantation because of impending loss of vascular access. Also, the high rate of referrals from other centers indicates that the presented procedure allows implantation of permanent central lines in situations, in which often there seems to be no other way. For these reasons we believe that the lack of a “control” group does not diminish the scientific value of our approach.

## Conclusions

Hybrid intervention technique combines an interventional approach with surgical catheter placement for establishing a permanent central venous line and providing vascular rehabilitation. Our data demonstrates the safe and highly successful use of this technique in children with chronic intestinal failure and end-stage vascular access. An individualized approach, sufficient expertise and the wide range of procedures described in this paper are required to access or reuse vessels in complex vascular situations. Progressive thoracic central venous obstruction, necessity for extended surgery and eventually for intestinal transplantation can be avoided or at least delayed with early use of the hybrid technique and consistent treatment of vascular stenosis and thrombosis.

## Data Availability Statement

The raw data supporting the conclusions of this article will be made available by the authors, without undue reservation.

## Ethics Statement

The studies involving human participants were reviewed and approved by the Institution's Internal Review Board. Written informed consent for interventions was obtained from the parents of all patients. Written informed consent from the participants' legal guardian/next of kin was not required to participate in this study in accordance with the national legislation and the institutional requirements.

## Author Contributions

LS, JH, and SW are responsible for conception and design of the study. LS provided the data from the hybrid interventions and drafted the manuscript. JH and FW provided data about the patients in the rehabilitation program. The authors agree to be accountable for all aspects of the work in ensuring that questions related to the accuracy or integrity of any part of the work are appropriately investigated and resolved. All authors revised this manuscript critically for important intellectual content and finally approved this version of the manuscript for submission.

## Funding

We acknowledge support by Open Access Publishing Fund of University of Tübingen.

## Conflict of Interest

The authors declare that the research was conducted in the absence of any commercial or financial relationships that could be construed as a potential conflict of interest.

## Publisher's Note

All claims expressed in this article are solely those of the authors and do not necessarily represent those of their affiliated organizations, or those of the publisher, the editors and the reviewers. Any product that may be evaluated in this article, or claim that may be made by its manufacturer, is not guaranteed or endorsed by the publisher.
